# Metabolic vulnerabilities of MYC-induced cancer

**DOI:** 10.18632/oncotarget.7223

**Published:** 2016-02-06

**Authors:** Arvin M. Gouw, Georgia G. Toal, Dean W. Felsher

**Affiliations:** Departments of Medicine and Pathology, Division of Oncology, Stanford University School of Medicine, CA, USA

**Keywords:** MYC, renal cell carcinoma, glutamine, RCC, hypoxia

All highly proliferating cells have two absolute requirements: abundant sources of energy and building blocks such as proteins, carbohydrates, and lipids. Specific gene products that coordinate massive induction of both energy and these chemical products are essential for normal organismal development and when constitutively activated would be anticipated to elicit uncontrolled growth or cancer. *MYC* is candidate gene product that can coordinate the transcription of genes that regulate energy and intermediary metabolism through its canonical targets through specific and more general mechanisms of gene binding, induction, and suppression [1, 2, 3]. Previous reports have noted MYC's role in the regulation of glucose and glutamine pathways to drive energy production. Further, MYC regulates nearly all of the central metabolic pathways in the cells, such as glycolysis, glutaminolysis, and nucleotide biosynthesis [1, 2, 3].

Recently, we have described a new transgenic mouse model of MYC-induced renal cancer (RCC) [4]. MYC is well known to be associated with aggressive RCC [4]. We found that these tumors exhibited marked alternations in glutamine metabolism. Indeed, inhibition of glutaminase with the inhibitor, BPTES (bis-2-(5-phenylacetamido- 1,2,4-thiadiazol-2-yl)ethyl sulfide), markedly impeded tumor growth [4]. Our results suggest that inhibiting glutaminolysis in RCC proves to be therapeutic. Human RCC may also exhibit altered glutamine metabolism in a subset of tumors. Targeting glutamine metabolism may be an effective approach for the treatment of at least some types of RCC.

Notably, we also found that MYC-induced RCC tumors exhibited other changes in metabolism, including marked altered production of specific phospholipids [4]. Hence, MYC may be altering the metabolic programming of not only glutamine and glucose, but also lipid metabolism, as has been suggested previously [5]. Understanding why and how these changes in metabolic programming occur may uncover additional therapeutic pathways that can be exploited to take advantage of unique vulnerabilities of MYC-induced tumors.

We propose that one unifying mechanistic explanation for our observations is that MYC changes metabolic programming to adapt cellular proliferation and growth under circumstances of marked hypoxic stress. This program that would be useful physiologically appears to be co-opted by MYC-associated cancer cells. Thus, MYC enables the ability to use both glucose and glutamine metabolism may generally reflect that under circumstances where energy and oxygen are in high demand, that glutamine is utilized precisely because it is abundant and can be used as both a source of carbons for metabolites and energy [6], which can be used to endure severe hypoxia.

The reason why glutamine is so critical under hypoxi conditions is glutamine can continue to participate tricarboxylic cycle (TCA). Hypoxia prevents glucose from entering the TCA via Hypoxia-inducible Factor (HIF1) that inhibits pyruvate conversion to acetyl-CoA, by shunting pyruvate to lactate via activation of both lactate dehydrogenase A (LDHA) and pyruvate dehydrogenase kinase (PDK1) that inhibits pyruvate dehydrogenase (PDH) [7]. Hence, a shift to glutamine from glucose as a source of energy and carbons appears to be a mechanism to bypass circumstances where glucose and/or oxygen are limiting. Finally, MYC-induced RCC are likely to be dependent upon both glutamine and glucose, while RCC associated with the genetic loss of Von Hippel Landau (VHL) are likely to depend more on glucose metabolism.

Finally, we observed that MYC-induced RCC exhibits a unique lipid profile of increased phosphoglycerides (PG), and phosphoinositols (PI). We believe this also can be explained by the requirements of glutamine as an energy source. Glutamine is not only an energy source via its catabolic processing in the Krebs Cycle, but also is a membrane lipid source for its highly proliferative phenotype. PG and PI are both important to maintain membrane production and integrity, but PI is also important for the production of signaling molecules which may in turn feedback to MYC.

**Figure 1:**
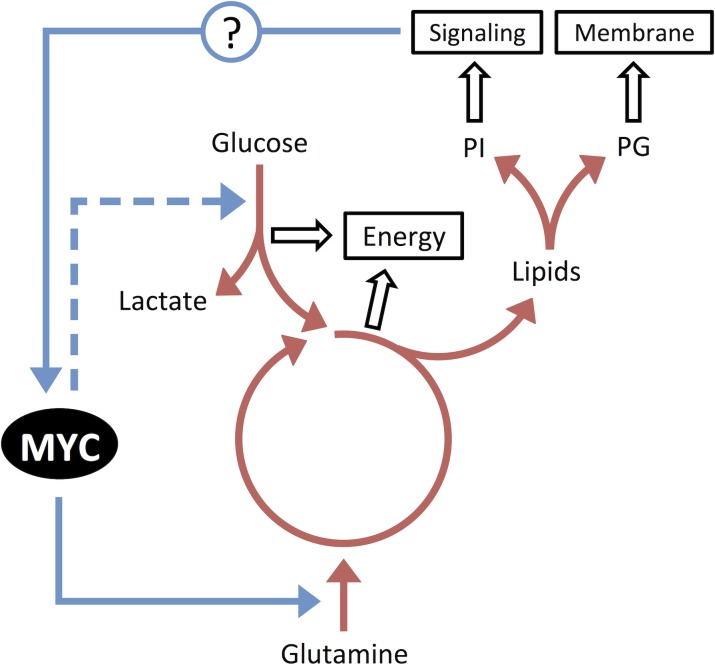
MYC can alter metabolic programming to shift from glucose to glutamine as an energy source in the Krebs cycle, resulting in a change in lipid production.

Hence, we propose a general model (Figure 1). MYC, by altering metabolic programming to shift from glucose to glutamine, necessarily results in a change in lipid production. Thus, MYC provides a way under stressful limiting conditions to not only generate energy but also metabolites required for sustained growth (Figure 1). Yet, by doing so also results in unique vulnerabilities. In MYC-dependent cancers, the inhibition of glutamine can have a dramatic influence on the ability of a tumor cell to grow and proliferate, as has now been experimentally shown for MYC-induced kidney and liver cancer [4].
